# Extractions of Medical Cannabis Cultivars and the Role of Decarboxylation in Optimal Receptor Responses

**DOI:** 10.1089/can.2018.0067

**Published:** 2019-09-23

**Authors:** Melissa M. Lewis-Bakker, Yi Yang, Rupali Vyawahare, Lakshmi P. Kotra

**Affiliations:** ^1^ Centre for Molecular Design and Preformulations, and Division of Experimental Therapeutics, Toronto General Research Institute, University Health Network, Toronto, Canada.; ^2^ Department of Pharmaceutical Sciences, Leslie Dan Faculty of Pharmacy, University of Toronto, Canada.; ^3^ Multi-Organ Transplant Program, Toronto General Hospital, Toronto, Canada.

**Keywords:** cannabinoid receptors, decarboxylation, extraction, medical cannabis, phytocannabinoids

## Abstract

**Introduction:** Phytocannabinoids, characteristic compounds produced by medical cannabis, interact with cannabinoid (CB) receptors (CB1 and CB2) as well as other receptor systems to exhibit their corresponding pharmacological effects. In their natural form, CBs such as Δ^9^-tetrahydrocannabinolic acid and cannabidiolic acid are inactive at these receptors, while their decarboxylated forms (Δ^9^-tetrahydrocannabinol and cannabidiol, respectively) are potent ligands at CB receptors. Thus, extraction and processing of medical cannabis for active constituents are important.

**Purpose and Methods:** Patients consuming medical cannabis often have limited alternative treatment options and in recent years, medical cannabis extracts have been popular as a substitute for dried cannabis plants, despite limited studies on these derivatives. We investigated three disparate cannabis cultivars and compared four chemical extraction methods head to head, viz. Soxhlet, ultrasound-assisted supercritical fluid, and microwave-assisted extractions, for their efficiency. We further characterized the chemical compositions of these extracts.

**Results:** Microwave extraction consistently produced completely decarboxylated phytocannabinoid extracts. Factors such as temperature and exposure time play important roles in the decarboxylation of phytocannabinoids, thereby generating pharmacologically active CBs, and these conditions may differ for each cannabis cultivar.

**Conclusion:** Chemical consistency and potency due to active compounds are in turn important in producing consistent and reliable medical cannabis extracts and their derivatives. These processes must be subject to higher levels of scientific rigor as the patient population around the world are seeking the help of such extracts for various clinical conditions, and as medical cannabis industry is receiving acceptance in various countries.

## Introduction

Numerous international and United Nations regulations served to control cannabis, and only in the recent years has the medicinal use of cannabis been again realized. Accordingly, the regulations are being reviewed and/or modified in this regard, thus allowing researchers to investigate the myriad of natural compounds present in cannabis in academic laboratories.^1,2,^^#^ Within the cannabis plant, at least 568 compounds have been identified to date, of which ∼120 are phytocannabinoids.^3,4^ Phytocannabinoids are biosynthesized from phenolic precursors in the cannabis plant, and several of these molecules carry a carboxylic acid moiety; major phytocannabinoid acids include Δ^9^-tetrahydrocannabinolic acid (Δ^9^-THCA, **1**), cannabidiolic acid (CBDA, **3**), and cannabigerolic acid (CBGA, **5**). Synthetic as well as plant-derived cannabinoids bind to cannabinoid (CB) receptors 1 and 2 (CB1 and CB2, respectively) in the central and peripheral tissues, and modulate these receptor responses for subsequent physiological effects.^5–7^

Acidic phytocannabinoids such as Δ^9^-THCA exhibit poor potency at CB receptors (cannabimimetic activity), whereas the decarboxylated phytocannabinoids such as Δ^9^-tetrahydrocannabinol (Δ^9^-THC), also referred to as neutral phytocannabinoids, exhibit high affinities and physiological activities.^8–11^ The decarboxylation step and the consistency of such decarboxylation are important to achieve reliable pharmacological effects when medical cannabis and its derivatives are used for their therapeutic efficacy.^12–14^ Patients typically smoke or vaporize the whole plant, or ingest the extract as edibles and infused edible oils. New industries are being fostered, focusing on medical cannabis extracts and their medical uses. In this context, cannabis extracts have become very popular in the recent months, including receiving U.S. Food and Drug Administration (FDA) approvals. For example, just a few months ago, Epidiolex^®^, a cannabis-extracted drug with >98% cannabidiol (CBD) and <0.5% of Δ^9^-THC, was approved by U.S. FDA for the treatment of intractable epilepsy including for pediatric use.^15–17^

There are conventional and domestic methods described for cannabis extraction such as ethanol extraction, maceration, butane extraction, and quick-wash alcohol extraction. Recently reported extraction methods include ultrasound-assisted extraction (UAE) and supercritical fluid extraction (SFE).^18–21^ Each method carries advantages and disadvantages depending on the compounds to be extracted, duration of extraction, temperature, and solvent, if any. It is typically desirable to use a solvent that solubilizes and carries compounds from the plant, and the temperature for extraction should minimize the loss of thermally labile groups or unwanted chemical transformations.

Soxhlet extraction involves continually extracting soluble phytochemicals from the plant under refluxing conditions of the solvent, typically ethanol. Soxhlet extraction may present few challenges such as duration of extraction, efficiency, and postextraction processing. Microwave-assisted extraction (MAE) employs microwaves to assist in the extraction of compounds from cannabis at elevated temperatures and pressures.^22–24^ The instantaneous energy transfer from solvent to biomass leads to a rapid increase in the temperature, and one can reach temperatures higher than the boiling point of the solvent if the pressure is contained.^25^ MAE offers additional advantages such as shorter extraction and reaction times, smaller quantities of solvent, and reproducibility.^25^ While all these methods help in extracting phytocannabinoids and other compounds from cannabis, activation of the extracts through the decarboxylation of phytocannabinoids remains a unique challenge.

**FIG. 1.**
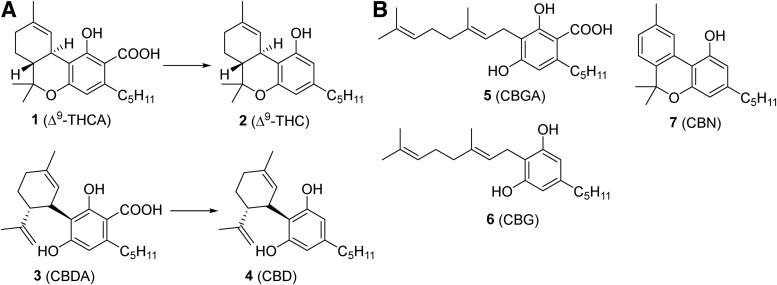
**(A)** Chemical structures of four major phytocannabinoids, Δ^9^-THCA (**1**), Δ^9^-THC (**2**), CBDA (**3**), and CBD (**4**), and their chemical transformation. **(B)** Chemical structures of CBGA (**5**), CBG (**6**), and CBN (**7**). Δ^9^-THC, Δ^9^-tetrahydrocannabinol; Δ^9^-THCA, Δ^9^-tetrahydrocannabinolic acid; CBD, cannabidiol; CBDA, cannabidiolic acid; CBG, cannabigerol; CBGA, cannabigerolic acid; CBN, cannabinol.

Decarboxylation of acidic phytocannabinoids could occur in an open or closed reactor. In an open reactor, decarboxylation of CBs has been demonstrated to occur at 37°C and 60°C after exposure for several hours or at 100°C for 60 min; in a closed reactor, the reaction could reach completion at 200°C in just 3 min.^26^ A disadvantage of using an open reactor for decarboxylation is that there is no agreeable temperature under atmospheric conditions at which efficient decarboxylation of the acidic CBs can occur without simultaneous evaporation of the solvent, along with any volatile compounds.^26^ Every phytocannabinoid carboxylate would have a different optimal condition for decarboxylation (Fig. 1), thus various medical cannabis cultivars with various chemical compositions would require different conditions to achieve complete decarboxylation of all phytocannabinoids.

A wide range of concentrations of Δ^9^-THC and CBD and their carboxylic acid precursors, Δ^9^-THCA and CBDA, are present in commercial medical cannabis products.^27,28^ If consumed, patients would be exposed to varying quantities of active phytocannabinoids leading to inconsistent physiological response. This could vary from batch to batch of the same product, as is often the case with plant-based products, providing highly uncertain and variable physiological effects. Due to the significant and renewed interest in medical cannabis extracts, their associated chemistry and biological activities, we undertook a comprehensive investigation to compare various chemical extraction protocols using three medical cannabis cultivars commercially sold in Canada. It is also important to note that medical cannabis is not a single substance, and is essentially classified into hundreds of varieties based on the composition of its phytocannabinoids. In this study, we explored three varieties of medical cannabis cultivars (balanced Δ^9^-THC/CBD, high CBD, and high Δ^9^-THC) with chemical distinction investigating their phytocannabinoid profiles, compared the efficiencies of extractions, chemical compositions, decarboxylation efficiencies of phytocannabinoid acids, and discussed their relevance to CB receptor responses. This study paves the way for further investigations into medical cannabis, relevant CBs, and the corresponding receptor responses.

## Experimental section

### Chemicals and reagents

Three *Cannabis sativa* L. dried flowers (cultivars **1**, **2**, and **3**), each with a different phytocannabinoid profile, were procured from the licensed producers. Milli-Q purified water and high-performance liquid chromatography grade methanol were used for chromatographic analyses. All other commercial solvents and reagents were used without further processing. Supercritical liquid CO_2_ (SFE grade) was purchased from Praxair^®^. Analytical standards for Δ^9^-THCA, CBDA, Δ^9^-THC, and CBD were purchased from Sigma-Aldrich^®^ as certified reference standards. Tissue culture media, fetal bovine serum, and trypsin were obtained from Gibco (MD, USA). HitHunter cAMP assay kit was purchased from DiscoverX Corporation (Fermont, CA, USA). All other chemicals were obtained from Sigma-Aldrich and were of analytical grade.

### Extractions

Medical cannabis samples were extracted using four chemical methods, each employing a different technology as described below. Medical cannabis used in each extraction experiment was in the range of 0.25–3.75 g. Yield of the extract was calculated using the formula: % Yield=(Weight of the extract)×100/(Weight of dried flower).

### Ultrasound-assisted extraction

An ultrasonic bath with 80 W of ultrasonic power, 63 W of heater power, and 40 kHz of operating frequency was used for UAE (sonication) experiments. Medical cannabis was weighed, then macerated using a mortar and pestle. Crushed plant material was then suspended in the solvent (10 mL/g), sonicated for 5 min at 25°C, and vacuum filtered using a sintered-glass funnel. An equivalent volume of solvent was added to the plant material, and the subsequent sonication–decantation–filtration steps were repeated for an additional two times. The combined filtrates were concentrated to dryness under reduced pressure at 25°C to afford a green sticky resin, which was stored at −8°C until further analyses.

### Supercritical fluid extraction

SFE was performed on a Jasco^®^ SFE/SFC system consisting of a fluid delivery module (CO_2_ pump and two solvent pumps), photodynamic array (PDA) detector, column oven, autosampler, fraction collector, and an automated back-pressure regulator. Medical cannabis was weighed, then macerated using a mortar and pestle, and was charged into a 10-mL extraction vessel. This vessel was placed in the column oven and subjected to extraction using supercritical CO_2_ as solvent A and ethanol as solvent B, at 25°C. The PDA detector was set at 254 nm, and the back-pressure regulator was set at 12 MPa. The flow rate was set to 10 mL/min for pumps 1 and 2 (solvents A and B, respectively) and 1 mL/min for the makeup pump. The gradient was programmed as follows: 0–25 min (100% A to 50% A/50% B, linear gradient); 25–26 min (50% A/50% B to 0% A/100% B, linear gradient), 26–30 (0% A/100% B to 100%A/0% B, linear gradient), and 30–30.2 min (100% A/0% B, isocratic), and the run time was 30.2 min. Extractions were performed three times consecutively on each sample until all phytocannabinoids were eluted from the biomass, as confirmed by PDA. All fractions were combined and were concentrated to dryness at 25°C to afford a green sticky resin, which was stored at −8°C until further analyses.

### Soxhlet extraction

Medical cannabis was weighed, then macerated using a mortar and pestle (crushed biomass) and transferred to a cellulose extraction thimble (43×123 mm; 2 mm thickness), and the thimble was inserted into Soxhlet extractor (size: 55/50). Ethanol (400 mL) was charged into the distillation flask, connected to the Soxhlet condenser, and subjected to refluxing for 4 h. Solvent extract was concentrated to dryness under reduced pressure at 25°C to afford a green sticky resin, which was stored at −8°C until further analyses.

### Microwave-assisted extraction

MAE was performed in a Biotage^®^ Initiator microwave (2.45 GHz, 400 W). Medical cannabis was weighed and powdered in a blender (Waring^®^ Laboratory) at 18,000 rpm for 4 sec or 22,000 rpm for 1 min. Powdered biomass was charged into microwave vials (20 mL size), ethanol (10–12 mL) and a stir bar were added, then the vial was capped and sealed. The biomass was first stirred at room temperature (RT) for 30 sec at 900 rpm, followed by microwave irradiation to maintain 120°C–170°C temperature for 20–45 min. Each extraction was performed in duplicate (at 3.75 g scale) or triplicate (at 1 g scale). The resulting suspension was then cooled to RT, filtered over Celite^®^, followed by a pad of activated carbon. The filtrate was concentrated under reduced pressure to dryness at 35°C to afford an orange sticky resin, which was stored at −8°C until further analyses.

### Analyses

The CB standards and cannabis extracts were analyzed using Waters^®^ Acquity™ ultra-performance liquid chromatography (UPLC) system equipped with Quaternary Solvent Manager, Sample Manager FTN, Acquity UPLC^®^ BEH column (2.1 mm×50 mm, C-18, 1.7 μm ID). The sample injection plate and the column were maintained at 15°C and 40°C, respectively. Mass spectra were recorded on a Waters MS 3100 mass spectrometer. Caffeine or Δ^9^-THC-*d*_3_ was used as an internal standard and was added to the injected samples to monitor the detector sensitivity of the UPLC-mass spectrometry (MS) system. Working stock solution of each CB standard was prepared using H_2_O/MeOH (3:7) with 0.1% formic acid (mobile phase I) and was appropriately further diluted with mobile phase I to obtain the standard curves. Each analytical sample was prepared by dissolving a defined amount of resin in mobile phase I, filtered (Millex-GV^®^ Syringe Filters, 0.22 μm; EMD Millipore), and further diluted with mobile phase I as needed. The internal standards were added and injected into the liquid chromatography-mass spectrometry (LC-MS) (injection volume, 2 μL [with caffeine] or 10 μL [with Δ^9^-THC-*d*_3_]). The mobile phase for LC-MS consisted of H_2_O (A) and MeOH (B), with 0.1% formic acid. The gradient was programmed as follows: 0–4.5 min (30% A/70% B to 0% A/100% B, linear gradient), 4.5–5.0 min (0% A/100% B isocratic), 5.0–5.2 min (0% A/100% B to 30% A/70% B, linear gradient), and 5.2–6 min (30% A/70% B isocratic). The flow rate was 0.6 mL/min, and all samples were analyzed in triplicate. The mass scan (150–500 m/z) and single ion recordings (SIRs) in positive and negative modes (+ve=287.20, 311.20, 315.23, 317.25, 359.23, 361.24; −ve=357.21 and 359.22 m/z) were monitored. SIR chromatograms were integrated using Empower3^®^ software, and the concentrations of the CBs were generated using the corresponding standard curves plotted in the Grafit^®^ software. Each phytocannabinoid concentration in the extract was computed using the formula:

\begin{align*}
& { \rm{ \% \ w / w \ Phytocannabinoid = }} \\ &\quad\left( {{ \rm{Phytocannabinoid \ concentration \ from \ Grafit \ fit}}} \right) \\ &\quad \times \left( {{ \rm{Dilution \ Factor}}} \right) \times { \rm{100 / }} \left( {{ \rm{Extract \ stock \ concentration}}} \right) { \rm.}
\end{align*}

### Receptor assays

The CBs Δ^9^-THC, Δ^9^-THCA, CBD, and CBDA were assayed against CB1 and CB2 receptors using cAMP assay platform with CHO-K1-CB1 and CHO-K1-CB2 cell lines, respectively (DiscoverX Corporation). Cells were cultured and maintained until further use. Incubation of the cells was carried out in a CO_2_ incubator at 37°C. All assays were conducted in triplicate. We measured the inhibition of adenylate cyclase and the production of cAMP when CB receptors are challenged with phytocannabinoids. Chemiluminescence was measured using Spectramax M5 plate reader. For data analyses, dose–response curves were generated with Graphpad^®^ Prism 7 software, and the EC_50_ values were determined using nonlinear regression curve fit.

### Agonist assays

About 30,000 cells/well were seeded into 96 well plates, and the plates were incubated overnight. The wells were decanted and 30 μL/well of phosphate-buffered saline (PBS) was added, followed by 15 μL of ligands solution containing test compound and forskolin (20 μM) in the PBS. Assay reactions were subjected to 30 min of incubation at 37°C, and 60 μL of cAMP detection working solution was added to each well. All wells were treated with 15 μL of anti-cAMP antibodies, and the plates were further incubated at RT for 1 h followed by the addition of 60 μL of solution A (DiscoverX kit) to each well.^29^ The plate was incubated for 3 h at RT, and chemiluminescence was counted using Spectramax M5 plate reader. CP-55940, a known full agonist for CB1 and CB2 receptors, was used as a positive control, and the activities of the tested samples were expressed as a percentage of the response to CP-55940.

### Antagonist assays

About 30,000 cells/well were seeded into 96 well plates, and the plates were incubated overnight. A 30 μL of PBS aliquot followed by test antagonist (7.5 μL at appropriate concentration) was added to each well, and the plate was incubated for 30 min. Then, 7.5 μL of ligands solution containing CP-55940 (EC_90_ concentration) and forskolin (20 μM) in PBS were added to each well, followed by further incubation for 30 min. Sixty microliters of cAMP detection working solution and 15 μL of anti-cAMP antibodies were then added to all wells. The plate was further incubated at RT for 1 h, after which 60 μL of solution A was added to all wells. Finally, the plate was incubated for 3 h at RT, and chemiluminescence readings from the wells were obtained on a plate reader. CB receptor antagonists, SR-141716 and AM630, were used as positive controls at CB1 and CB2 receptors, respectively. Receptor responses to the tested compounds are expressed as a percentage of the response to CP-55940. cAMP accumulation at 100% represents response of the receptor in the absence of compound, and that at 0% represents cAMP accumulation in the presence of highest concentration of CP-55940 (1 μM) reflecting full agonism at the CB receptor.

## Results and Discussion

As discussed above, CBs interact with the CB receptors, as well as other receptors such as 5-HT_1A_ to exert their activities.^7–10^ In various ligand binding studies, binding constants (*K*_i_) for Δ^9^-THC (**2**) and CBD (**4**) have been described in the range of 5.05–80.3 nM and 4.35–10 μM, respectively, for CB1 receptors, and 3.13–75.3 nM and 2.4–10 μM, respectively, for CB2 receptors.^10,30–39^ Δ^9^-THCA (Fig. 1A) is shown to have measurable, but very weak binding at both human CB1 and human CB2 receptors, equating to approximate *K*_i_ values of 3.1 and 12.5 μM, although the certified reference standard of Δ^9^-THCA used in the aforementioned study was found to be contaminated with 2% Δ^9^-THC.^9^ We also observed 2% Δ^9^-THC as a contaminant in the Δ^9^-THCA certified reference standard. We could not find any data on CBDA binding to CB1 and CB2 receptors; however, we expected that it may not bind to either receptor. It is also important to note that when several ligands are present at the same time in proximity of a receptor, ligands binding to ligand binding sites on the receptor could produce a completely different functional response than they would individually. For example, Δ^9^-THC binds to the orthosteric site whereas CBD is an allosteric ligand to CB receptors, and their functional responses as individual ligands could be different from that when they bind to the receptor simultaneously.

**FIG. 2.**
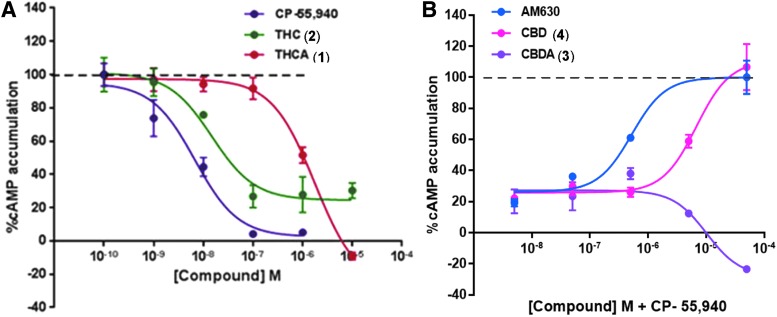
Dose–response curves for Δ^9^-THCA (**1**) and Δ^9^-THC (**2**) as agonists at CB1 receptor **(A)**, and for CBDA (**3**) and CBD (**4**) as antagonists at CB2 receptor **(B)**. CB, cannabinoid.

We assessed the receptor efficacy of Δ^9^-THC, Δ^9^-THCA, CBD, and CBDA at human CB1 and CB2 receptors by measuring the functional response of adenylate cyclase activity, to verify the idea that THCA and CBDA are less potent than their corresponding decarboxylated products (Fig. 2; Table 1). The ligand binding site (orthosteric vs. allosteric) is taken for granted based on literature evidence, the functional response was measured by quantifying the response of adenylate cyclase (inhibition of adenyl cyclase-catalyzed mediated cAMP production).^5–7^ In these studies, Δ^9^-THC elicited a more potent response in comparison with Δ^9^-THCA, as did CBD in comparison with CBDA. In case of Δ^9^-THCA, it is almost three orders of magnitude weaker than Δ^9^-THC (EC_50_ 1.8±0.7 μM vs. 15±1 nM) at CB1 receptor as an agonist (Fig. 2A; Table 1), which is significant. CBDA is more than 10-fold weaker than CBD (EC_50_ >100 μM vs. 7±2 μM) at CB2 receptor as an antagonist, where interestingly CBD itself is a weak ligand with micromolar potency (Fig. 2B; Table 1). The potency of CBD as an antagonist at CB2 receptor is in agreement with results published by Martinéz-Pinalla et al.^40,41,^^†^ All of these observations buttress the idea that phytocannabinoids consistency is vital for the resulting activities of cannabis extracts, and decarboxylation of acidic phytocannabinoids will determine majority of the potency of such extracts.

**Table 1.** Cannabinoid Receptor Activities of Major Phytocannabinoids

Compound	CB1			CB2		
	Mode of action	Efficacy (%)	EC_50_ (μM)	Mode of action	Efficacy (%)	EC_50_ (μM)
CP-55940	Agonist	100	0.0025±0.0009	Agonist	100	0.0026±0.0004
AM-630	—	—	—	Antagonist	100	0.146±0.084
SR-141716	Antagonist	129	0.024±0.003	—	—	—
Δ^9^-THC	Partial agonist	60	0.015±0.001	Weak partial agonist	20	>10
Δ^9^-THCA	Weak agonist	100	1.8±0.7	Weak agonist	100	30±15
CBD	Antagonist	40	>10	Antagonist	100	7±2
CBDA	NA	0	>10	NA	−23	>10

CP-55940 was used as a reference agonist in CB1 receptor assays, SR-141716 as a reference antagonist in CB1, and AM-630 was used as a reference antagonist in CB2 receptor assays.

Δ^9^-THC, Δ^9^-tetrahydrocannabinol; Δ^9^-THCA, Δ^9^-tetrahydrocannabinolic acid; CB, cannabinoid; CBD, cannabidiol; CBDA, cannabidiolic acid; NA, no activity.

As medical cannabis and cannabis extracts gain ground as “legitimate medical substances” in several territories around the world, including North America, chemical consistency and associated biological activities are critical for pharmaceutical grade products and for patient care. In this vein, we have employed four distinct chemical methods, viz. UAE, SFE, Soxhlet extraction, and MAE, to investigate the yields and decarboxylation potential in the context of active phytocannabinoids in the extracts, using three different commercial medical cannabis cultivars.

### Ultrasound-assisted extraction

**FIG. 3.**
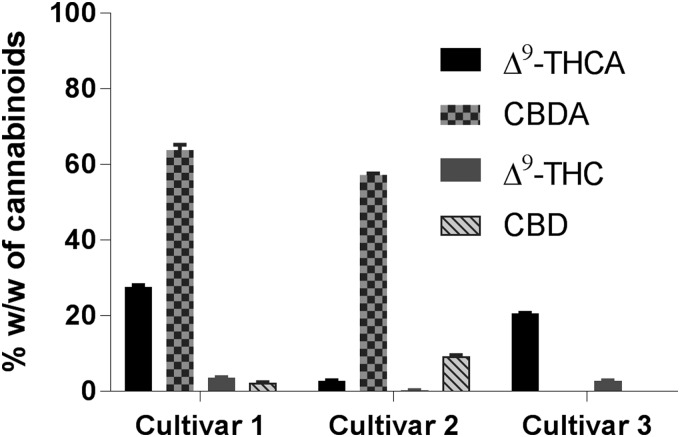
CBs content in the extracts of medical cannabis cultivars employing UAE in ethanol. UAE, ultrasound-assisted extraction.

Three medical cannabis cultivars were extracted employing UAE, and the corresponding yields for the resins, and of acidic and neutral CBs were determined (Table 2). Solvent systems with different polarities, that is, hexanes as a nonpolar solvent, ethanol as a polar solvent and isopropanol/hexanes (1:1) were employed. Hexanes would be more effective in extracting lipophilic compounds while the alcohols are expected to favor more polar compounds. Cultivar **1** was subjected to UAE using these three solvents. UAE using hexanes and ethanol yielded similar quantity of extracts but lower than that obtained from isopropanol/hexanes solvent system (Table 2). UPLC-MS analysis of the extracts showed an inverse relationship between the yield of the extract and CB content. In comparison with the other two solvent systems, the extract obtained from UAE using 1:1 isopropanol/hexanes had the lowest percentage of total CBs (Table 2). These higher yields of cannabis extracts due to isopropanol/hexanes solvent system may be partially due to higher quantities of non-CB compounds when compared with the extracts using the other two solvent systems. In addition, ethanol is a very well-accepted solvent for pharmaceutical manufacturing processes. Thus, subsequent UAE experiments were performed in ethanol to maximize the phytocannabinoid yields. Cultivars **2** and **3** were extracted using ethanol employing UAE. Upon comparison, cannabis extract from cultivar **3** was obtained with the highest yield, but it had the lowest quantity of phytocannabinoids among the three cannabis cultivars (Table 2; Fig. 3).

**Table 2.** Yields and Cannabinoids Content in the Extracts of Medical Cannabis Cultivars Employing Ultrasound-Assisted Extraction

Solvent	Cultivars	% Yield	Cannabinoid (w/w %) in the extract			
			Δ^9^-THCA	CBDA	Δ^9^-THC	CBD
Hexanes	Cultivar 1	24	22.0±0.4	56.6±1.8	4.7±0.3	8.9±0.4
Isopropanol/hexanes (1:1)	Cultivar 1	48	18.1±0.3	21.1±0.2	3.8±0.1	5.5±0.1
Ethanol	Cultivar 1	30	27.6±0.4	63.8±1.4	3.6±0.1	2.3±0.1
	Cultivar 2	24	2.8±0.1	57.2±0.4	0.2±0.1	9.3±0.2
	Cultivar 3	40	20.5±0.2	0^a^	2.8±0.1	0^a^

^a^ Analyte is below the lower limit of quantitation (LLQ). (Supplementary Table S1).

Chemical analyses of the cannabis extracts revealed higher concentrations of the major phytocannabinoid acids, that is, Δ^9^-THCA and CBDA, and smaller amounts of the corresponding decarboxylated compounds (Δ^9^-THC and CBD). These results suggested that UAE alone is effective in extracting primarily phytocannabinoids in their acid form from the dried cannabis but could not accomplish efficient decarboxylation.

### Supercritical fluid extraction

**FIG. 4.**
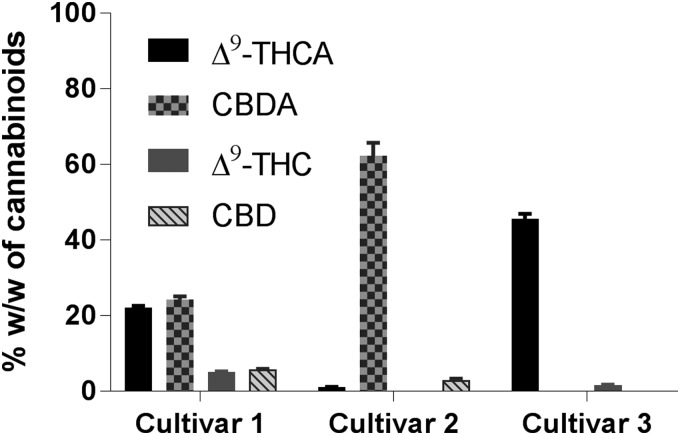
CBs content in the extracts of medical cannabis cultivars employing SFE. SFE, supercritical fluid extraction.

A summary of the yields for major phytocannabinoids in cannabis resin using SFE method is presented in Table 3. Three solvent systems were employed during SFE; solvent system 1 involved soaking the plant material in a mixture of supercritical liquid CO_2_ and ethanol (1:1) inside the extraction vessel for 5 min, then extracting the plant material using isocratic conditions at 12 MPa pressure. Solvent system 2 is a modified method of Omar et al.,^19^ which allowed the removal of terpenes efficiently using supercritical CO_2_ and ethanol (0–20% ethanol), where ethanol permits the extraction of higher quantities of phytocannabinoids. Solvent system 3 is a combination of solvent systems 1 and 2, where the gradient elution using supercritical CO_2_ in ethanol (100–0%) should extract both nonpolar and polar compounds from dried cannabis. These solvent systems did not significantly influence the extraction yields for cultivar **1** (22–26% yield; Table 3); the total amounts of CBs in the resin were the highest for solvent system 1 and lowest for solvent system 2 (Table 3). Solvent system 3 closely represents the conditions for elution under supercritical conditions, and was used for all subsequent experiments. Similar yields of the extracts from cannabis cultivars **1** and **3** were obtained using solvent system 3, and these were higher than that obtained from cannabis cultivar **2** (Table 3; Fig. 4). In addition, the total percentages of CBs in the extracts were highest in the extract from cannabis cultivar **2** (Table 3). An LC-MS analysis of the cannabis extracts obtained using SFE shows smaller quantities of Δ^9^-THC and CBD than the corresponding acidic phytocannabinoids.

**Table 3.** Yields and Cannabinoids Content in the Extracts of Medical Cannabis Cultivars Employing Supercritical Fluid Extraction

Solvent system^a^	Cultivars	% Yield	Cannabinoid (w/w %) in the extract			
			Δ^9^-THCA	CBDA	Δ^9^-THC	CBD
1	Cultivar 1	22	18.8±0.6	68.8±1.5	5.4±0.1	8.2±0.3
2	Cultivar 1	26	18.2±0.6	10.2±1.4	5.0±0.2	6.6±0.1
3	Cultivar 1	28	22.1±0.4	24.2±0.8	5.1±0.1	5.8±0.1
	Cultivar 2	15	1.1±0.1	62.3±3.4	0^b^	2.9±0.4
	Cultivar 3	26	45.5±1.4	0^b^	1.6±0.1	0^b^

^a^ Solvent A=supercritical CO_2_ and solvent B=ethanol; (a) solvent system 1: 5 min static (soaking the plant material) with 50% A/50% B, 25 min dynamic with 50% A/50% B, acquisition time=30 min; (b) solvent system 2: 15 min dynamic with 100% A, acquisition time=15 min followed by 30 min dynamic with 80% A/20% B, acquisition time=30 min; (c) solvent system 3: 0.1–25 min: 100% A/0% B to 50% A/50% B, 25–26 min: 100% B, 26–30 min: 100% A, acquisition time=30 min.

^b^ Analyte is below the LLQ (Supplementary Table S1).

### Soxhlet extraction

**FIG. 5.**
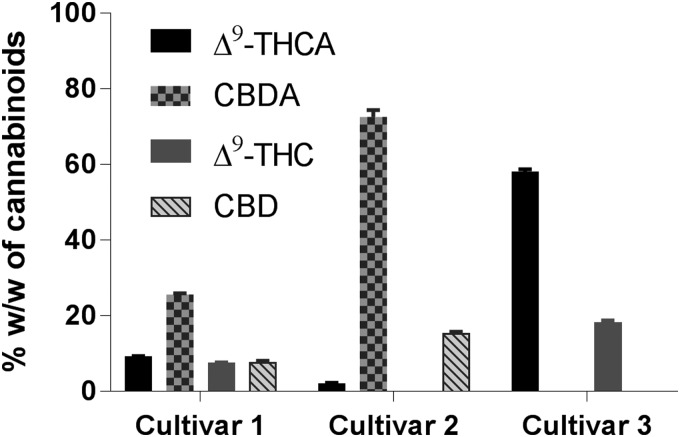
CBs content in the extracts of medical cannabis cultivars employing Soxhlet extraction in ethanol.

Table 4 and Figure 5 summarize the yields of acidic and neutral CBs in cannabis resin extracted using Soxhlet extraction. Using ethanol exclusively as the solvent, the highest yields for the extracts were observed for cultivars **1** and **3**, and similar to those with SFE, yield was lowest for cannabis cultivar **2** (31% vs. 21%; Table 4). However, the total percentage of phytocannabinoids in the cannabis extract was highest for cultivar **2**, and was lowest for cultivar **1** (Table 4). Soxhlet extraction was carried out under refluxing conditions using ethanol, and these conditions due to thermal energy are expected to promote decarboxylation of Δ^9^-THCA, CBDA, and other acidic phytocannabinoids into the corresponding decarboxylated forms.

**Table 4.** Yields and Cannabinoids Content in the Extracts of Medical Cannabis Cultivars Employing Soxhlet Extraction with Ethanol

Cultivars	% Yield of resin	Cannabinoid (w/w %) in the extract			
		Δ^9^-THCA	CBDA	Δ^9^-THC	CBD
Cultivar 1	31	9.2±0.1	25.5±0.4	7.5±0.1	7.8±0.2
Cultivar 2	21	2.0±0.2	72.5±1.9	0^a^	15.4±0.3
Cultivar 3	31	58.1±0.6	0^a^	18.3±0.4	0^a^

^a^ Analyte is below the LLQ (Supplementary Table S1).

Chemical analyses of the cannabis extracts from all three cultivars showed a higher percentage of CBs Δ^9^-THC and CBD than those employing UAE or SFE (Table 4 vs. Tables 2 and 3). One must note that the decarboxylation occurred only partially, and there is a significant amount of natural phytocannabinoid acids Δ^9^-THCA and CBDA still present in the cannabis extracts (Table 4; Fig. 5). In contrast, previously published results of the Soxhlet extraction of hemp seeds (reported by us)^22^ and that of dried plant material by Wianowska et al.^42^ reported higher yields of neutral CBs, possibly due to the conditions employed. Although similar extraction conditions (extractor volume, time, and type/volume of solvent) for hemp seeds were employed, the difference in results may be attributed to the much lower quantity of Δ^9^-THCA in the hemp seed to begin with. Hence, in the same time frame, there is less Δ^9^-THCA to be carboxylated. In the latter case with dried plant material, different conditions involving a smaller 100-mL extractor (vs. 200 mL), methanol and hexanes as the solvents (vs. ethanol), lower solvent volumes (75 mL vs. 400 mL) were used. The larger solvent volume used in our extraction produces more dilute conditions, which may subsequently affect decarboxylation rates. This dilution effect may also account for the absence of cannabinol (CBN) in the Soxhlet extracts, also in contrast to reports by Wianowska et al.,^42^ since based on the biosynthetic pathway of major phytocannabinoids,^43^ CBN is produced from Δ^9^-THC.

### Microwave-assisted extraction

Cannabis was suspended in ethanol and was subjected to higher temperatures with stirring to explore the potential for the extraction of phytocannabinoids and simultaneously facilitate the decarboxylation of naturally occurring acidic phytocannabinoids, Δ^9^-THCA and CBDA, into Δ^9^-THC and CBD, respectively.^22^ Temperatures >130°C accomplished the extraction and decarboxylation at respectable levels; that is, >99% of decarboxylation of acidic phytocannabinoids.

**FIG. 6.**
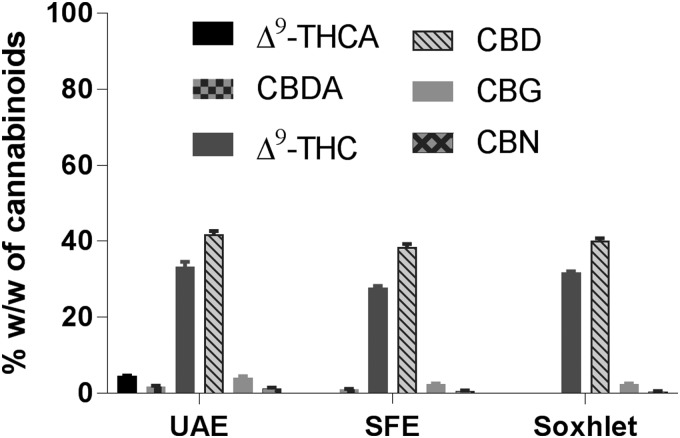
CBs content in the UAE, SFE, or Soxhlet extracts of medical cannabis cultivar **1** subjected to microwave-assisted heating at 150°C for 10 min.

The extracts of cultivar **1**, isolated from UAE, Soxhlet, and SFE methods, were dissolved in ethanol and were subjected to microwave irradiation at 150°C for 10 min, to facilitate decarboxylation reaction. As expected, there was a decrease in the acidic CBs and a corresponding increase in the respective neutral counterparts (Table 5; Fig. 6). Only the extract from Soxhlet was completely decarboxylated within the 10-min microwave irradiation, likely due to the earlier partial decarboxylation, and extracts from SFE and UAE required longer time to achieve complete decarboxylation. When the extracts were subjected to the temperatures of 150°C, minor quantities of cannabigerol (CBG) and CBN were observed in the range of 0.5–4.2% (Table 5; Fig. 6 and Supplementary Figs. S7A–D and S8A–D).

**Table 5.** Comparison of the Yields of Medical Cannabis Extracts and Cannabinoids (%) from Cultivar 1 Without and With Subsequent Heating (at 150°C for 10 min) subsequent to Ultrasound-Assisted Extraction, Supercritical Fluid Extraction, or Soxhlet Extraction Methods

	Extraction method	% Yield	Cannabinoid (w/w %)					
			Δ^9^-THCA	CBDA	Δ^9^-THC	CBD	CBG	CBN
Before heating	UAE (ethanol)	30	27.6±0.4	63.8±1.4	3.6±0.1	2.3±0.1	0^b^	0^b^
	SFE^a^	28	25.1±0.9	52.8±1.8	5.1±0.1	5.8±0.1	0^b^	0^b^
	Soxhlet	31	9.2±0.1	25.5±0.4	7.5±0.1	7.8±0.2	0^b^	0^b^
Postheating (150°C for 10 min)	UAE (ethanol)	76	4.6±0.1	1.9±0.1	33.4±1.2	41.9±0.8	4.2±0.3	1.4±0.1
	SFE^a^	77	0^b^	1.1±0.1	27.8±0.5	38.5±0.8	2.5±0.1	0.7±0.1
	Soxhlet	83	0^b^	0^b^	31.9±0.2	40.2±0.6	2.5±0.1	0.5±0.1

^a^ Solvent system 3: 0.1–25 min: 100% A/0% B to 50% A/50% B, 25–26 min: 100% B, 26–30 min: 100% A, acquisition time=30 min.

^b^ Analyte is below the LLQ (Supplementary Table S1).

SFE, supercritical fluid extraction; UAE, ultrasound-assisted extraction; CBG, cannabigerol; CBN, cannabinol.

**FIG. 7.**
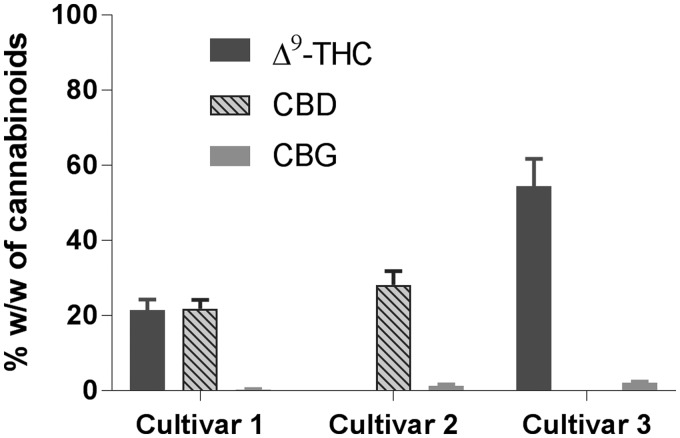
CBs content in the extracts of medical cannabis cultivars employing microwave-assisted extraction at 150°C for 20 min in ethanol.

We then investigated the potential to extract and decarboxylate dried plant material directly within the microwave reactor. Thus, dried plant material suspended in ethanol was subjected to heating with stirring in a microwave reactor, in triplicate using ∼1 g of plant material in ethanol at 150°C for 20 min (Table 6; Fig. 7). One must note that plant material was macerated and charged into the microwave directly for extraction, with no additional step for extraction. Interestingly, consistent yields with complete decarboxylation of the phytocannabinoids were accomplished in one step following this process (Table 6; Fig. 7). Chemical analyses indicated that there were no acid forms of phytocannabinoids such as Δ^9^-THCA and CBDA in the resulting extract, and complete decarboxylation was achieved yielding Δ^9^-THC and CBD (Table 6; Fig. 7). Interestingly, depending on the type of cultivar, variable amount of CBG, up to 2.1%, was observed in the extract derived using MAE (Table 6). Extraction yield was in the range of 19.6–24.4% for the concentrated resin.

**Table 6.** Yields for Resins (%) and Cannabinoids (w/w %) Using Microwave-Assisted Extraction Conducted at 150°C for 20 Min

Cultivars	% Yield of extract	Cannabinoid (w/w %)		
		Δ^9^-THC	CBD	CBG
Cultivar 1	22.5±0.3	21.4±2.8	21.8±2.3	0.3±0.1
Cultivar 2	19.6±0.4	0	28.1±3.7	1.3±0.3
Cultivar 3	24.4±1.3	54.4±7.3	0^a^	2.1±0.3

^a^ Analyte is below the LLQ (Supplementary Table S1).

**FIG. 8.**
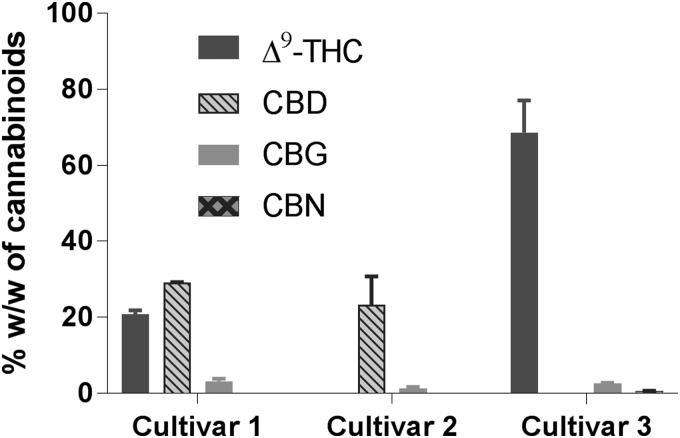
CBs content in the extracts of medical cannabis cultivars employing microwave-assisted extraction at 150°C for 30 min in ethanol.

Any changes to the quantities of dried cannabis (3.7 g vs. 1 g) required slightly modified conditions for the extraction and decarboxylation, such as subjecting the plant material to 150°C for up to 30 min or at 140°C for up to 45 min achieved slightly higher yields for the extracts, as well as complete decarboxylation (Table 7; Fig. 8). The yields for extracts from cultivars **1** and **3** were higher than that for cultivar **2** (28.1% and 32.0% vs. 20.8%). The amount of decarboxylated phytocannabinoids, Δ^9^-THC and CBD, in the extracts was found to be lowest for cultivar **2** and highest for cultivar **3** (Table 7). An interesting observation is that a small quantity of CBN (0.6%) was observed in the extract from cultivar **3** cannabis (Supplementary Figs. S7E and S8E). This particular cultivar of the plant had minimal quantity of naturally produced CBDA and a high quantity of Δ^9^-THCA. When this cultivar was subjected to MAE process for only 20 min (1 g scale), we did not detect any CBN.

**Table 7.** Yields (w/w) for Resins and Phytocannabinoids Using Microwave-Assisted Extraction Conducted at 150°C for 30 Min

Cultivars	% Yield of resin	Cannabinoid (w/w %)			
		Δ^9^-THC	CBD	CBG	CBN
Cultivar 1	28.1±1.7	20.9±1.0	29.2±0.1	3.2±0.7	0^a^
Cultivar 2	20.8±1.5	0^a^	23.4±7.4	1.3±0.4	0^a^
Cultivar 3	32.0±3.6	68.6±8.4	0^a^	2.7±0.1	0.6±0.1

^a^ Analyte is below the LLQ (Supplementary Table S1).

CBN (**7**) is an oxidation product of Δ^9^-THC (**2**) and is typically observed during prolonged storage of dried cannabis plant material with exposure to heat, light, air, or acidic conditions.^44–46^ The presence of CBN in the extracts obtained after microwave irradiation may be due to longer exposure of the extracts and plant material to higher temperatures because at shorter exposure time, we did not observe any CBN (Table 6 vs. Table 7).^47^ Transformation of Δ^9^-THC into CBN may occur through either a radical mediated^48,49^ or an oxidation of Δ^9^-THC to CBN through epoxy and hydroxylated intermediates, a plausible means in the current experiments.^50^

We also observed that CBG (**6**) was generated in the range of 1.3–3.2% (w/w) when decarboxylation of phytocannabinoids was performed using MAE (Table 7). The amount of CBG was higher when there was higher proportion of Δ^9^-THC in the extract, and it was lower when CBD quantities were higher in the extract. Within the plant, CBGA is the precursor for the biosynthesis of Δ^9^-THCA and CBDA. Hanuš et al. proposed a mechanism earlier for the synthesis of THCA from CBGA,^51^ which could lead to CBG postdecarboxylation. Further studies are necessary to confirm this mechanism and the conditions that promote the transformation of Δ^9^-THC and CBD into CBG.

## Summary

Medical cannabis extracts are gaining popularity around the world in the recent years, and many countries are accommodating access to these plant materials for hundreds of thousands of patients. It is well recognized that acidic phytocannabinoids are inactive at CB receptors, and their decarboxylated analogs are potent ligands. Four common extraction methodologies, UAE, SFE, Soxhlet, and MAE, were employed to obtain extracts from three cultivars of medical cannabis. While UAE and SFE extract the acidic CBs in their natural forms since heating is not employed, Soxhlet and MAE allow for the conversion of acidic CBs into their neutral active forms through decarboxylation.

MAE proves to be a superior method when extraction and decarboxylation of phytocannabinoids have to be achieved because of the possibility to apply controlled temperatures, shorter extraction times, and reproducibility. MAE also has the potential to be employed for preparative and industrial scale of production, due to ready scalability. The ability to completely decarboxylate acidic CBs to pharmacologically active CBs in various cultivars of cannabis is important to manufacture quality products with measurable potency for use by patients.

LLD/LLQ for phytocannabinoids analyses, LC/MS and mass spectra profiles (Supplementary Figs. S1, S2, S3, S4, S5, S6) for the cannabis extracts are found in Supplementary Data.

## Supplementary Material

Supplemental data

Supplemental data

Supplemental data

Supplemental data

Supplemental data

Supplemental data

Supplemental data

Supplemental data

Supplemental data

## Abbreviations Used

Δ^9^-THC: Δ^9^-tetrahydrocannabinol
Δ^9^-THCA: Δ^9^-tetrahydrocannabinolic acid
CB: cannabinoid
CBD: cannabidiol
CBDA: cannabidiolic acid
CBG: cannabigerol
CBGA: cannabigerolic acid
CBN: cannabinol
ESI: electron spray ionization
FDA: Food and Drug Administration
LC-MS: liquid chromatography-mass spectrometry
LLD: lower limit of detection
LLQ: lower limit of quantitation
MAE: microwave-assisted extraction
MS: mass spectrometry
NA: no activity
PBS: phosphate-buffered saline
RT: room temperature
SFE: supercritical fluid extraction
SIR: single ion recording
UAE: ultrasound-assisted extraction
UPLC-MS: ultra-performance liquid chromatography-mass spectrometry

## Notes

^#^ S.A. Ross, pers. comm.

^†^ There are some experimental differences in the receptor assays between our protocols and those in Ref. 40, such as the use of a transient cell line (vs. stable cell line), different reference agonist (JWH133 vs. CP-55940) and a lower concentration of forskolin (0.5 μM vs. 20 μM. But overall, these do not alter the final interpretations significantly.
