# Supplementary material for: Extractions of Medical Cannabis Cultivars and the Role of Decarboxylation in Optimal Receptor Responses
Source: Cannabis Cannabinoid Res. 2019 Sep 23;4(3):183–94. doi: 10.1089/can.2018.0067 (PMC6757234; doi:10.1089/can.2018.0067)
Supplement: Supplemental data [file Supp_Data1-Table1.pdf]

Supplementary Data

Liquid Chromatography-Mass Spectrometry Analyses and Lower Limit of Detection/Lower Limit of Quantitation

Instruments: Waters Acquity H-Class UPLC with Waters 3100 Mass spectrometer

Column: Acquity UPLC BEH column (2.1 mm × 50 mm, C-18, 1.7 μm ID)

Mobile phase: H_2O (A) and MeOH (B), with 0.1% formic acid as follows: 0–4.5 min (30% A/70% B to 0%A/100% B, linear gradient), 4.5–5.0 min (0% A/100% B isocratic), 5.0–5.2 min (0% A/100% B to 30% A/70% B, linear gradient), and 5.2–6 min (30% A/70% B isocratic).

Flow rate: 0.6 mL/min

Temperature: Sample injection plate = 15°C; column = 40°C

Ionization: Electrospray, positive (+ve) and negative (–ve)

Scan range: (150–500 m/z)

Mass single ion recordings: +ve = 287.20, 311.20, 315.23, 317.25 m/z; –ve = 357.21 and 359.22 m/z

Supplementary Table S1. LLD and LLQ Values for the Phytocannabinoids in This Study

Phytocannabinoid	LLD (ng/mL)	LLQ (ng/mL)
Δ^9-THC	1.0	2.5
Δ^9-THCA	1.0	2.5
CBD	2.5	5.0
CBDA	1.0	2.5
CBN	6	12
CBG	59	76

Δ^9-THC, Δ^9-tetrahydrocannabinol; Δ^9-THCA, Δ^9-tetrahydrocannabinolic acid; CBD, cannabidiol; CBDA, cannabidiolic acid; CBG, cannabigerol; CBN, cannabinol; LLD, lower limit of detection; LLQ, lower limit of quantitation.
