# Supplementary material for: Extractions of Medical Cannabis Cultivars and the Role of Decarboxylation in Optimal Receptor Responses
Source: Cannabis Cannabinoid Res. 2019 Sep 23;4(3):183–94. doi: 10.1089/can.2018.0067 (PMC6757234; doi:10.1089/can.2018.0067)
Supplement: Supplemental data [file Supp_Fig7.pdf]

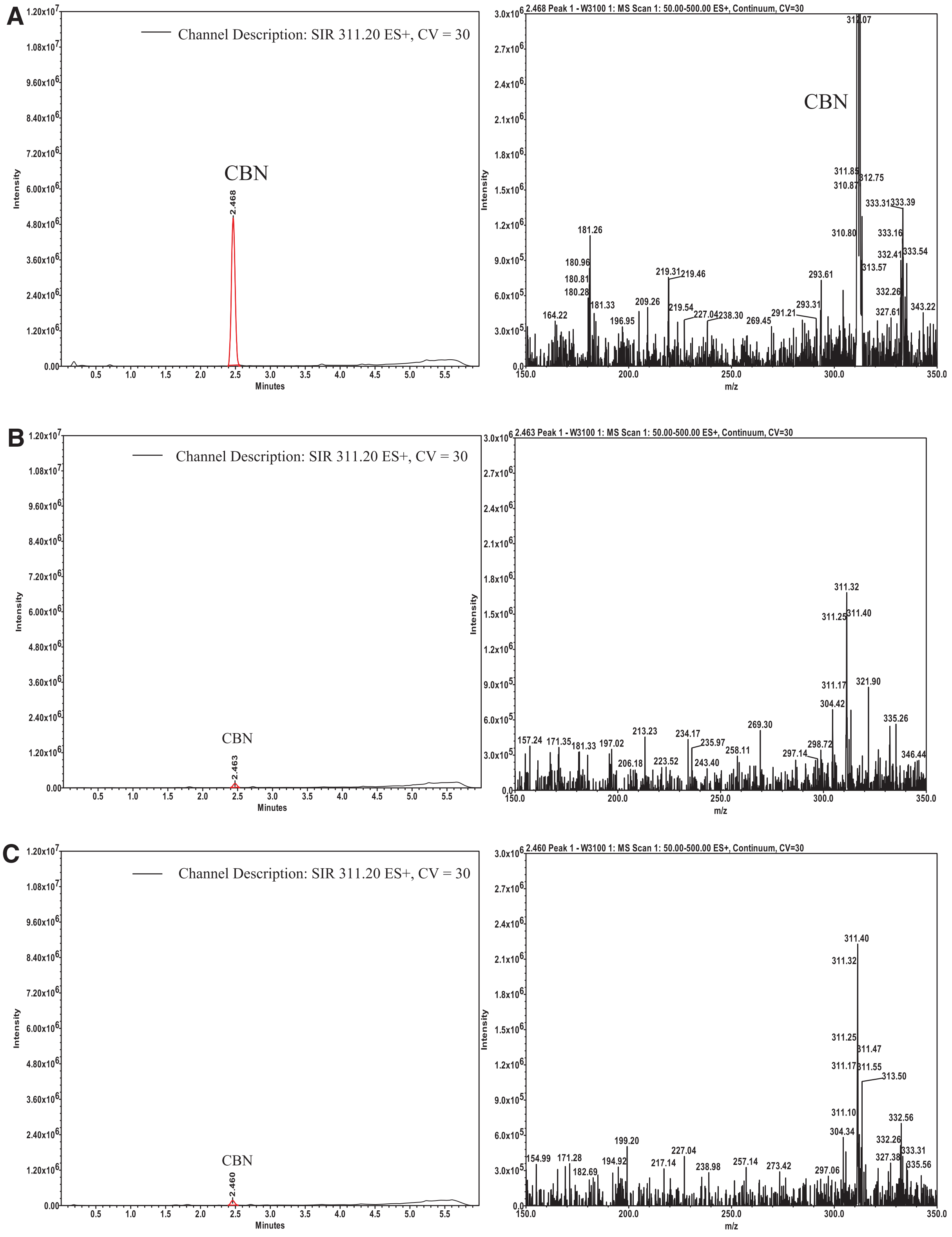


**SUPPLEMENTARY FIG. S7.** Comparison of representative mass chromatograms (left panel) and mass spectra (right panel) using ESI (+ve) mode SIR at 311.20 Da (m/z, $MH^+$) for CBN reference standard **(A)**; Strain 1 extracts first subjected to UAE **(B)** or SFE **(C)** or Soxhlet **(D)** method, followed by microwave heating; **(E)** cultivar 3 extract obtained using MAE. SFE, supercritical fluid extraction. CBN, cannabinol.

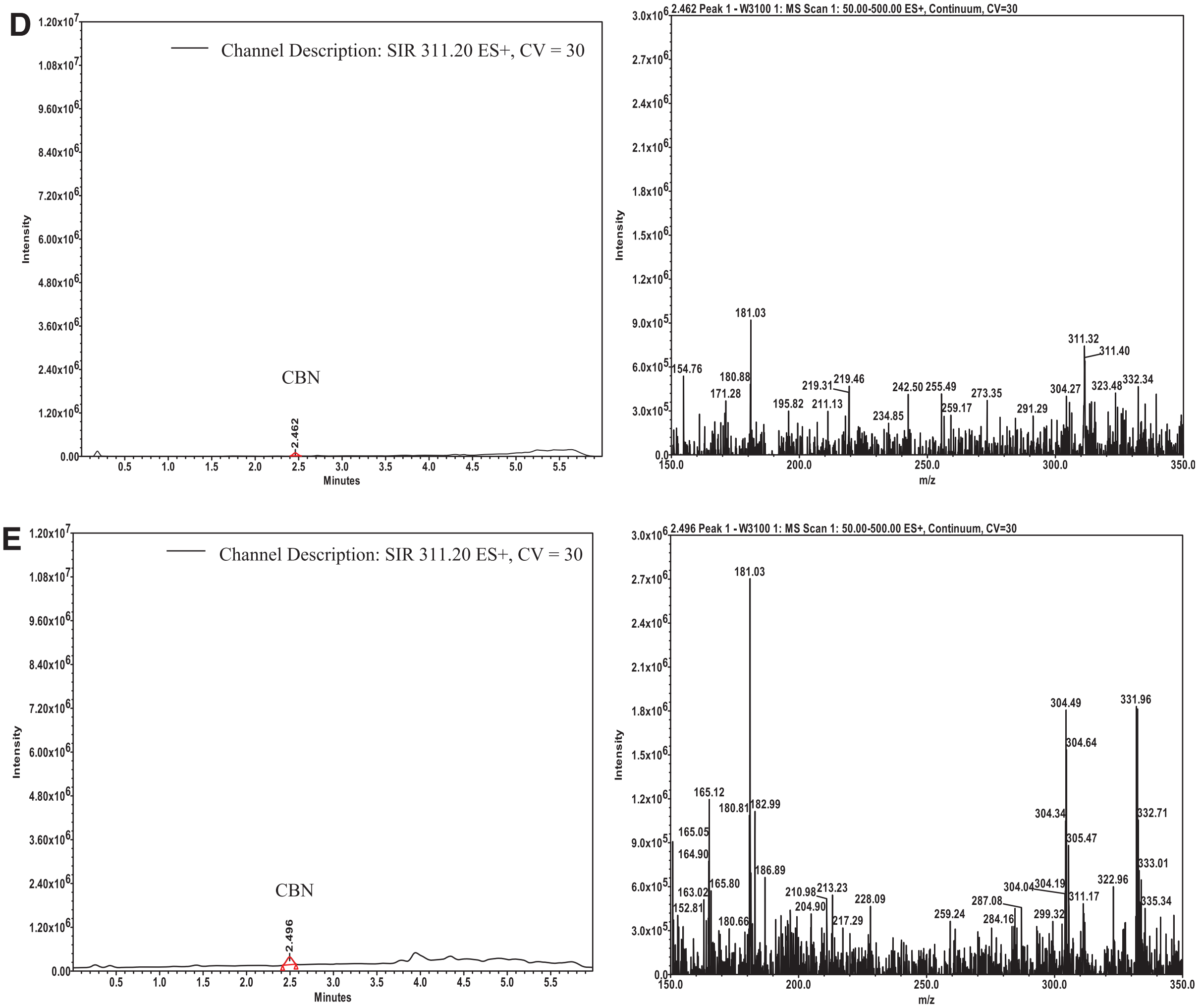


**SUPPLEMENTARY FIG. S7.** (*Continued*)
