# Supplementary material for: Extractions of Medical Cannabis Cultivars and the Role of Decarboxylation in Optimal Receptor Responses
Source: Cannabis Cannabinoid Res. 2019 Sep 23;4(3):183–94. doi: 10.1089/can.2018.0067 (PMC6757234; doi:10.1089/can.2018.0067)
Supplement: Supplemental data [file Supp_Fig8.pdf]

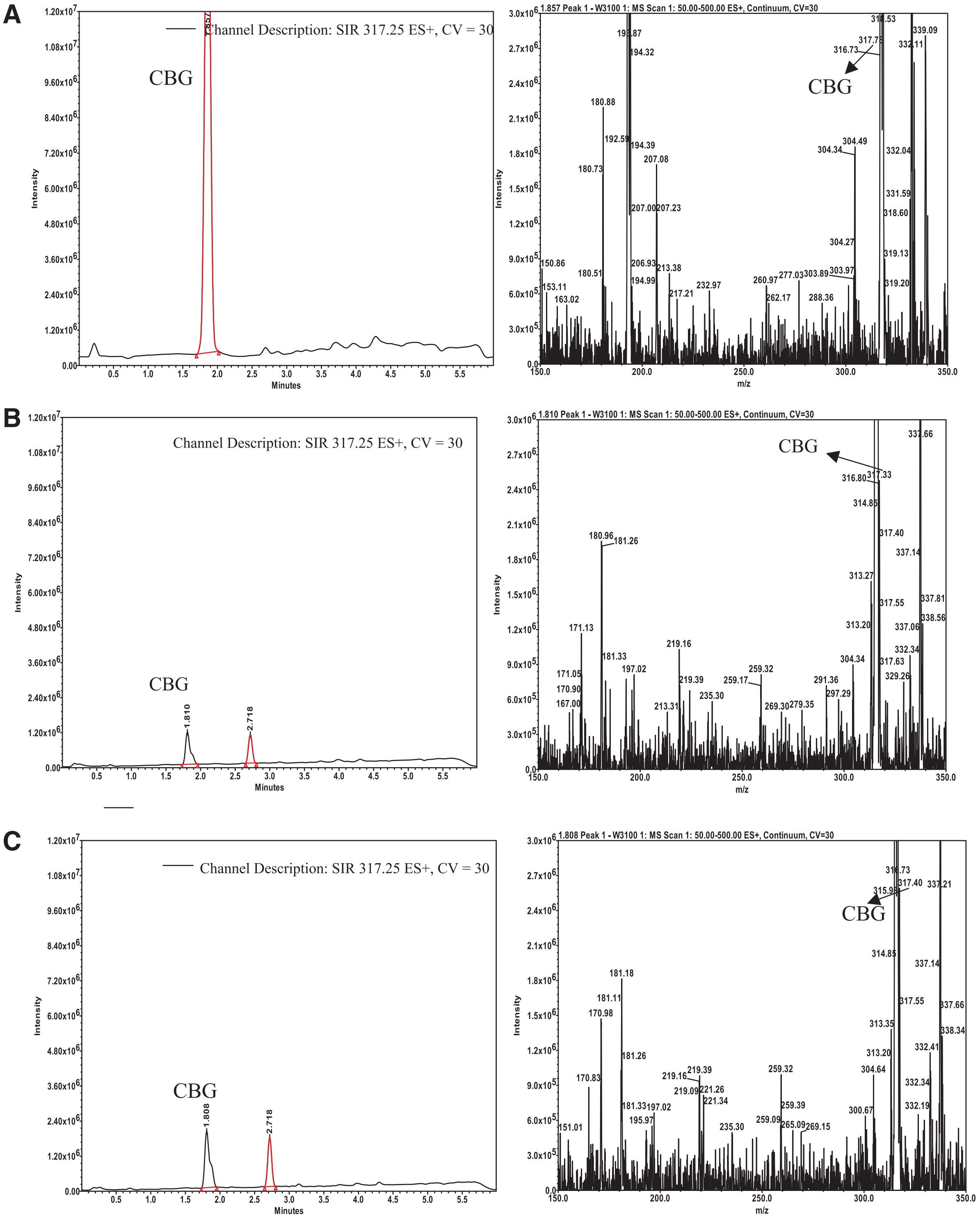

SUPPLEMENTARY FIG. S8. Comparison of representative mass chromatograms (left panel) and mass spectra (right panel) using ESI (+ve) mode SIR at 317.25 Da (m/z, MH^+) for **(A)** CBG reference standard; Strain 1 extracts using **(B)** UAE or **(C)** SFE or **(D)** Soxhlet method followed by microwave heating; **(E)** Strain 3 extract obtained using MAE. CBG, cannabigerol.

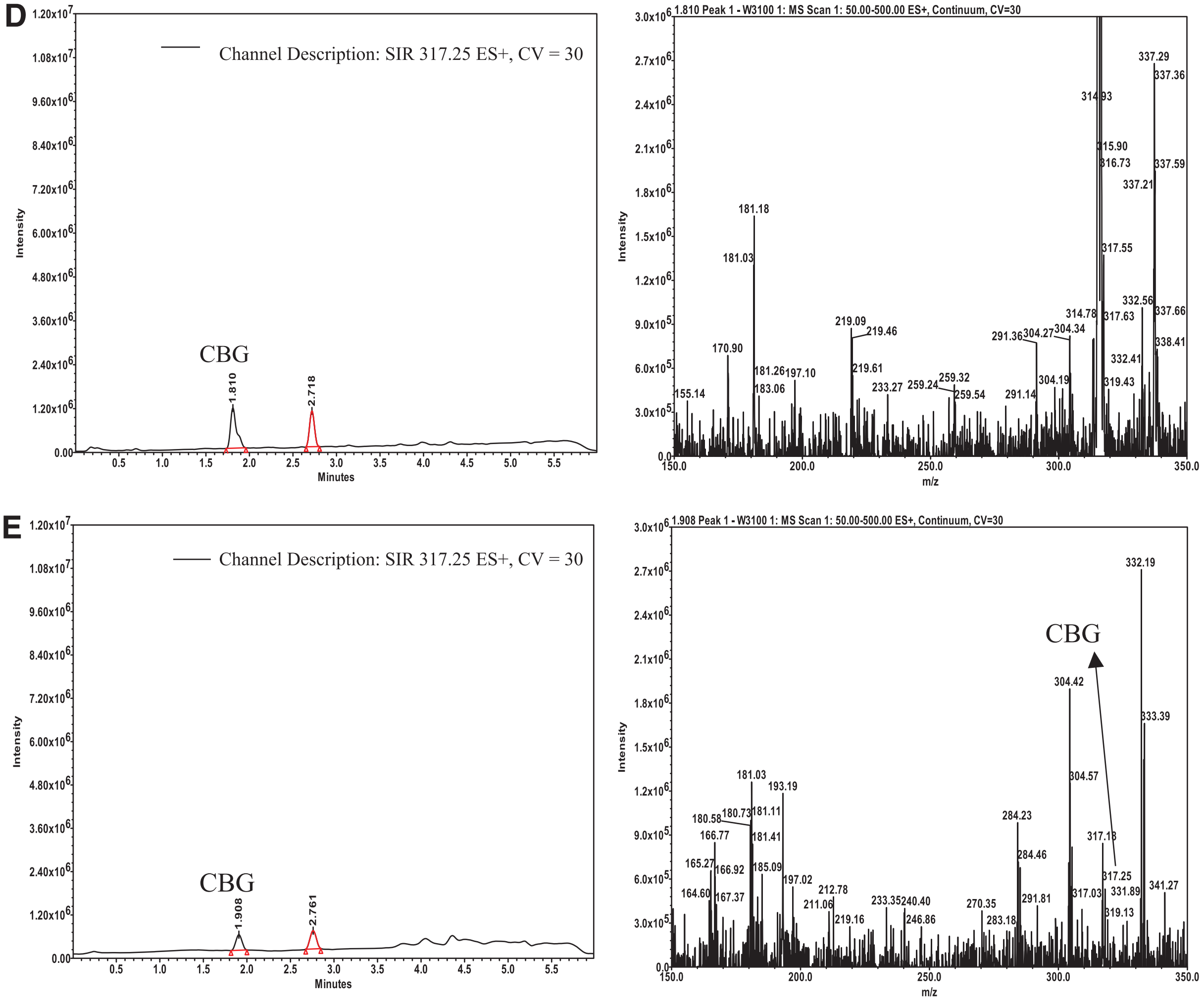

SUPPLEMENTARY FIG. S8. (*Continued*)
