# Supplementary material for: Extractions of Medical Cannabis Cultivars and the Role of Decarboxylation in Optimal Receptor Responses
Source: Cannabis Cannabinoid Res. 2019 Sep 23;4(3):183–94. doi: 10.1089/can.2018.0067 (PMC6757234; doi:10.1089/can.2018.0067)
Supplement: Supplemental data [file Supp_Fig2.pdf]

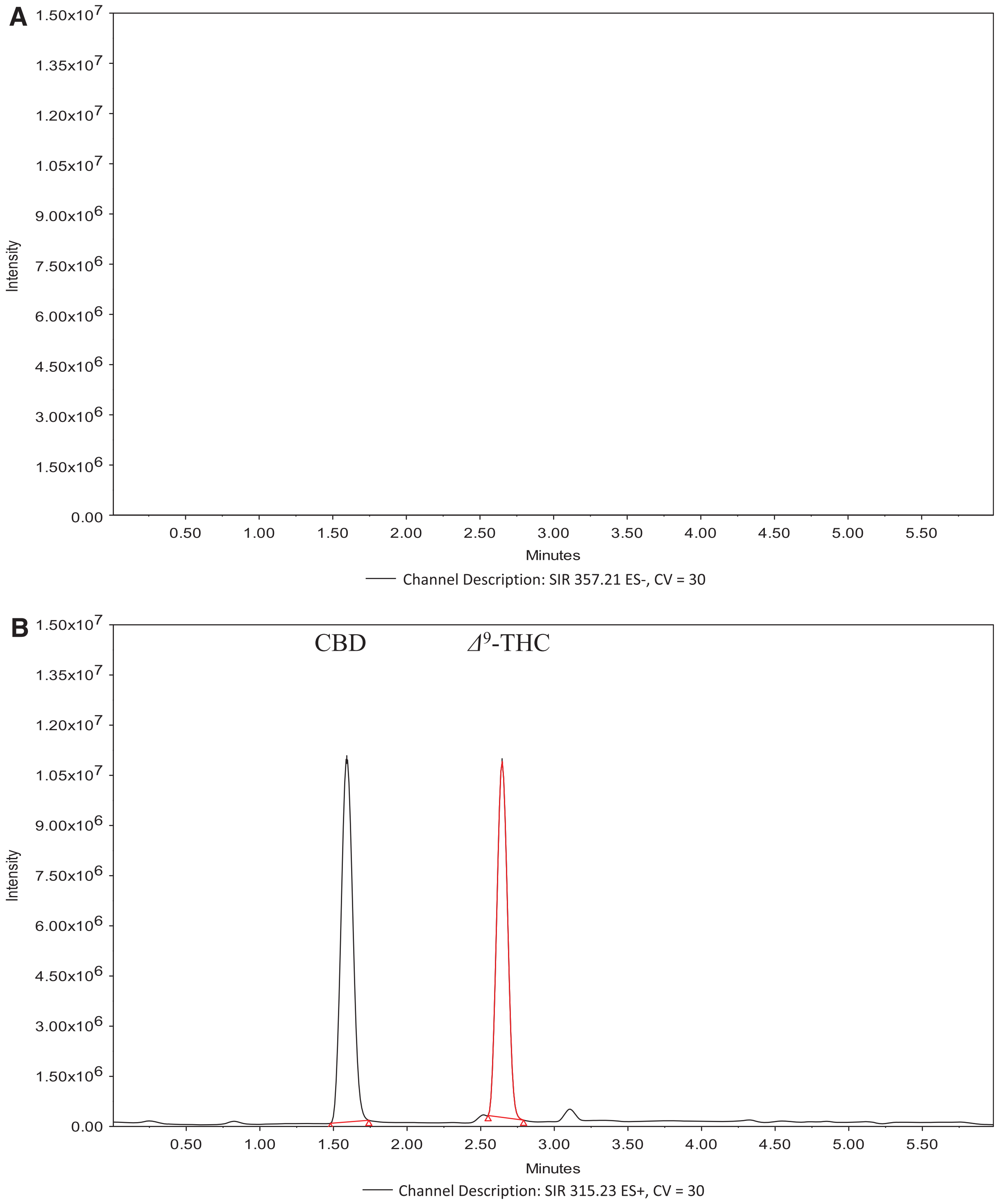

SUPPLEMENTARY FIG. S2. Representative mass chromatograms for cultivar 1 extract using MAE. **(A)** ESI (–ve) mode for detection of acidic phytocannabinoids and **(B)** ESI (+ve) mode for detection of neutral phytocannabinoids. MAE, microwave-assisted extraction.
