# Supplementary material for: Extractions of Medical Cannabis Cultivars and the Role of Decarboxylation in Optimal Receptor Responses
Source: Cannabis Cannabinoid Res. 2019 Sep 23;4(3):183–94. doi: 10.1089/can.2018.0067 (PMC6757234; doi:10.1089/can.2018.0067)
Supplement: Supplemental data [file Supp_Fig6.pdf]

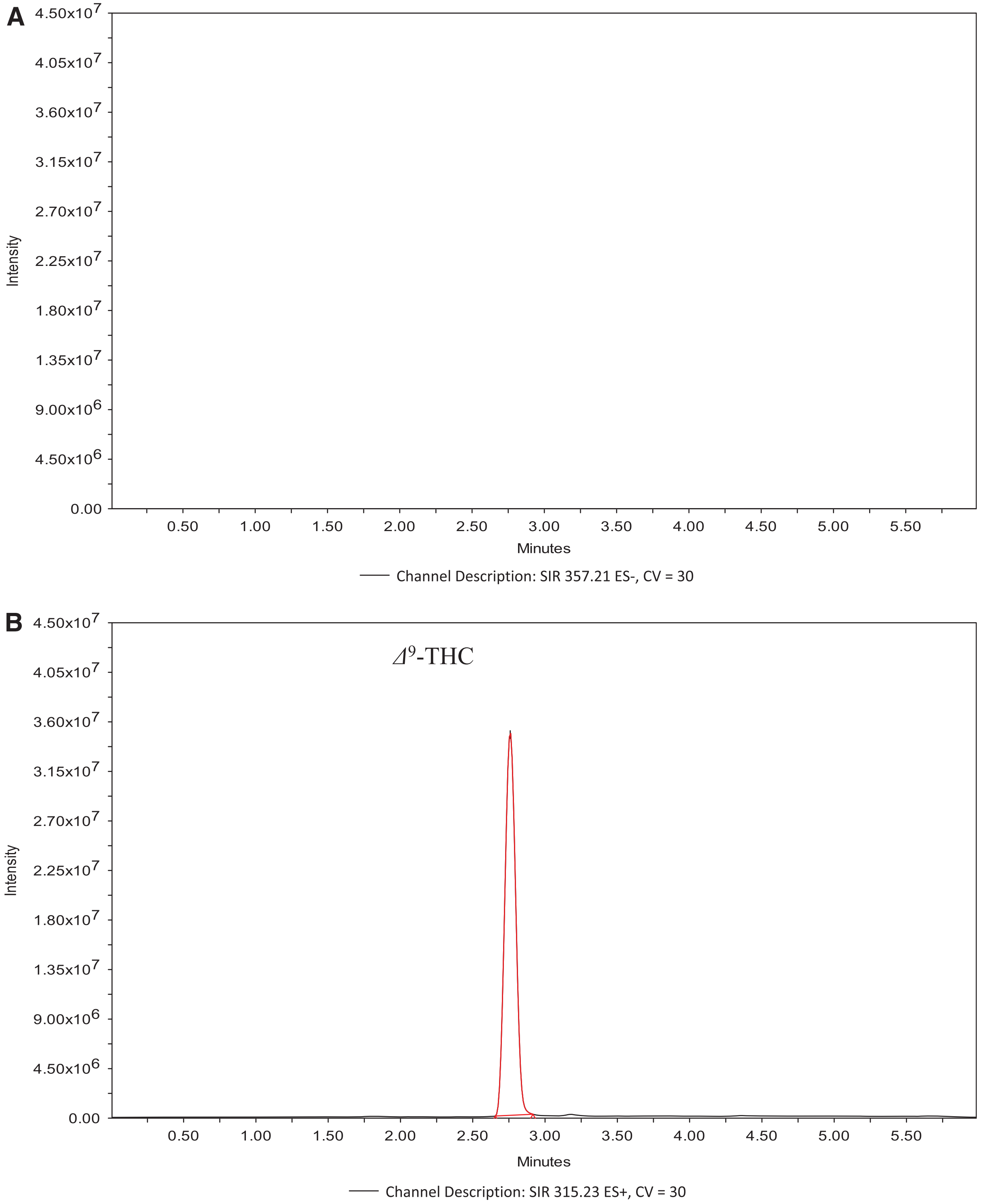

SUPPLEMENTARY FIG. S6. Representative mass chromatograms for cultivar 3 extract using MAE. **(A)** ESI (–ve) mode for detection of acidic phytocannabinoids, and **(B)** and **(C)** ESI (+ve) mode for detection of neutral phytocannabinoids at SIR 315.23 and 317.25 Da, respectively; SIR, single ion recording.

C

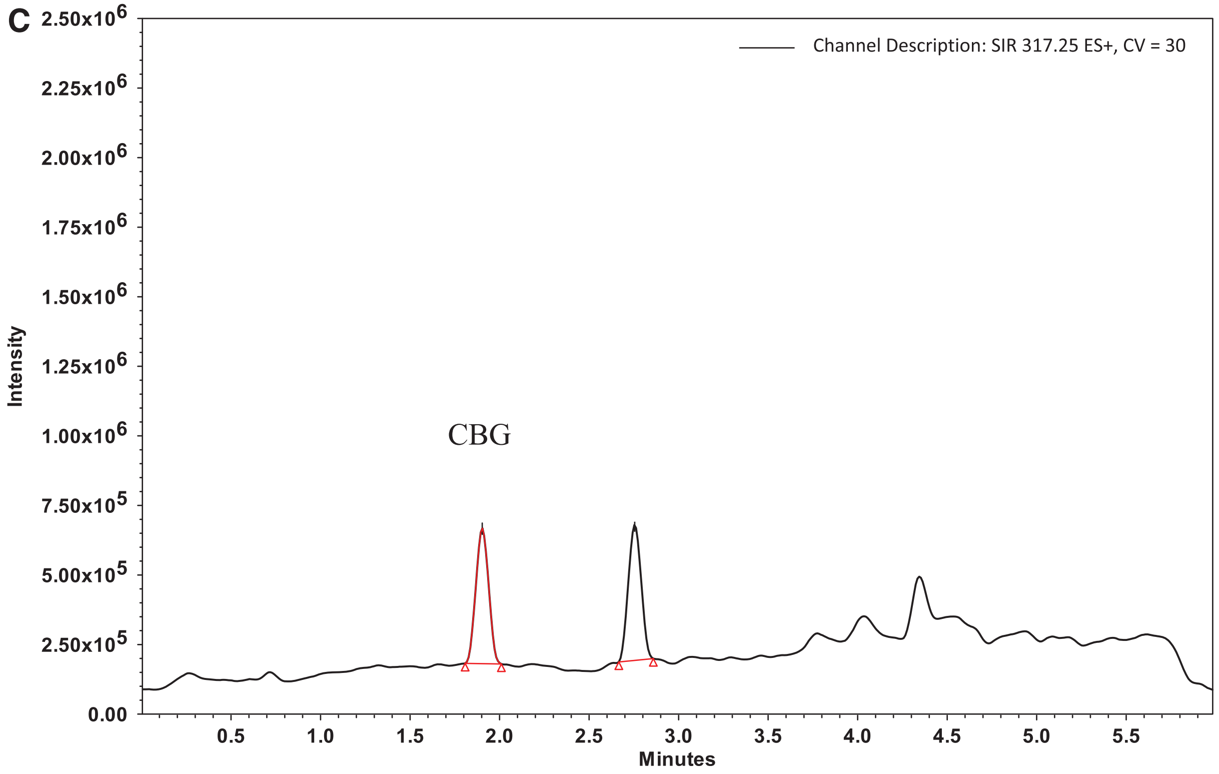

SUPPLEMENTARY FIG. S6. (*Continued*)
